# Supplementary material for: Evaluation of Liquid Organic Acids on the Performance, Chyme pH, Nutrient Utilization, and Gut Microbiota in Broilers under High Stocking Density
Source: Animals (Basel). 2023 Jan 12;13(2):257. doi: 10.3390/ani13020257 (PMC9854823; doi:10.3390/ani13020257)
Supplement: Supplementary file 1 [file animals-13-00257-s001.zip › Table S1.pdf]

Table S1. Effects of organic acids on the alpha diversity of cecal bacteria in broilers under high stocking density in grower phase [1].

Items	CON + NSD	CON + HSD	OA + NSD	OA + HSD	SEM	*p* - Value		
						OA	Density	INT
Sobs	404.67	388.33	398.67	407.50	5.555	0.572	0.747	0.285
Shannon	4.10	3.49	3.95	4.12	0.149	0.426	0.468	0.204
Simpson	0.06	0.15	0.10	0.08	0.020	0.754	0.425	0.175
Chao	431.22	417.70	431.53	441.06	4.850	0.237	0.839	0.25

[1] Data represent the means of six replicates (n = 6). CON, control group; OA, organic acids group; NSD, normal stocking density; HSD, high stocking density; SEM, standard error of the means; INT, interaction.
