# Supplementary material for: Evaluation of Liquid Organic Acids on the Performance, Chyme pH, Nutrient Utilization, and Gut Microbiota in Broilers under High Stocking Density
Source: Animals (Basel). 2023 Jan 12;13(2):257. doi: 10.3390/ani13020257 (PMC9854823; doi:10.3390/ani13020257)
Supplement: Supplementary file 1 [file animals-13-00257-s001.zip › Table S2.pdf]

Table S2. Effects of organic acids supplementation on cecal microbial composition at phylum level in broilers under high stocking density in grower phase (%) [1].

Items	CON + NSD	CON + HSD	OA + NSD	OA + HSD	SEM	*p* - Value		
						OA	Density	INT
Firmicutes	69.46	76.01	81.36	76.02	2.581	0.266	0.909	0.267
Bacteroidota	28.79	22.76	17.12	22.09	2.652	0.265	0.923	0.319
Cyanobacteria	0.39	0.51	0.49	0.59	0.068	0.533	0.452	0.947
Actinobacteriota	0.65	0.39	0.65	0.53	0.098	0.745	0.352	0.733
other	0.71	0.33	0.38	0.78	0.124	0.813	0.972	0.142
Firmicutes/Bacteroidetes	2.91	8.25	9.46	4.38	1.435	0.642	0.965	0.081

[1] Data represent the means of six replicates (n = 6). CON, control group; OA, organic acids group; NSD, normal stocking density; HSD, high stocking density; SEM, standard error of the means; INT, interaction.
