# Supplementary material for: Evaluation of Liquid Organic Acids on the Performance, Chyme pH, Nutrient Utilization, and Gut Microbiota in Broilers under High Stocking Density
Source: Animals (Basel). 2023 Jan 12;13(2):257. doi: 10.3390/ani13020257 (PMC9854823; doi:10.3390/ani13020257)
Supplement: Supplementary file 1 [file animals-13-00257-s001.zip › Table S3.pdf]

Table S3. Effects of organic acids supplementation on cecal microbial composition at genus level in broilers under high stocking density in grower phase (%) [1].

Items	CON + NSD	CON + HSD	OA + NSD	OA + HSD	SEM	*p* - Value		
						OA	Density	INT
Christensenellaceae_R-7_group	1.67	3.43	4.57	4.24	0.48	0.051	0.433	0.254
Enterococcus	3.71	0.39	4.22	1.37	1.22	0.769	0.234	0.926
Norank_f__Eubacterium_coprostanoligenes_group	1.35	0.86	1.29	1.52	0.21	0.504	0.773	0.414
Blautia	2.30	1.23	2.96	0.83	0.29	0.798	0.004	0.301
Norank_f__norank_o__Clostridia_vadinBB60_group	4.69	2.49	5.05	4.03	0.6	0.44	0.196	0.631
Bacteroides	5.03	1.88	2.92	3.99	0.54	0.999	0.325	0.053
Unclassified_f__Lachnospiraceae	3.56	2.72	4.26	2.84	0.3	0.493	0.069	0.626
Norank_f__norank_o__Clostridia_UCG-014	5.23	4.42	8.63	6.65	0.81	0.091	0.39	0.715
Lactobacillus	14.24	32.2	20.93	18.85	3.83	0.669	0.313	0.207
Norank_f__norank_o__RF39	1.25	0.77	2.64	1.47	0.23	0.011	0.04	0.365
Subdoligranulum	2.56	1.84	3.49	2.84	0.44	0.299	0.459	0.969
Barnesiella	3.36	11.56	0.55	2.73	2.22	0.198	0.249	0.498
Ruminococcus_torques_group	1.94	2.17	2.11	2.91	0.28	0.432	0.373	0.629
Alistipes	17.02^{a}	5.72^{b}	9.59^{ab}	9.85^{ab}	1.44	0.514	0.038	0.031
Rikenella	1.05	1.02	1.21	2.21	0.26	0.195	0.348	0.322
Streptococcus	1.3	4.93	1.3	2.81	1.27	0.692	0.341	0.692
Parabacteroides	1.65	2.01	1.57	1.67	0.46	0.836	0.822	0.893
Unclassified_f__Oscillospiraceae	1.71	0.9	1.32	2.93	0.31	0.172	0.494	0.107
Norank_f__Ruminococcaceae	2.59	1.74	1.96	3.68	0.45	0.474	0.634	0.166

UCG-005	1.06	1.44	1.68	3.96	0.4<br>4	0.0<br>61	0.10<br>8	0.2<br>44

[1] Data represent the means of six replicates (n = 6). [a, b] Means in the same row without the same superscripts differ significantly (p < 0.05). CON, control group; OA, organic acids group; NSD, normal stocking density; HSD, high stocking density; SEM, standard error of the means; INT, interaction.
